# Use of Psychological Interventions by Healthcare Workers in Post-COVID-19 Period: Longitudinal Study

**DOI:** 10.1192/j.eurpsy.2025.1271

**Published:** 2025-08-26

**Authors:** B. García-Vázquez, C. Bayón Pérez, M. F. Bravo Ortiz, R. Mediavilla Torres

**Affiliations:** 1 Psychiatry, Clinical Psychology and Mental Health, Hospital Universitario La Paz (HULP); 2 Psychiatry, Instituto de Investigación Sanitaria La Paz (IdiPAZ); 3 Psychiatry, Universidad Autónoma de Madrid (UAM); 4 Centro de Investigación Biomédica en Red de Salud Mental (CIBERSAM); 5 Instituto de Investigación Carlos III; 6 Hospital Universitario La Princesa; 7 Instituto de Investigación Sanitaria del Hospital Universitario La Princesa, Madrid, Spain

## Abstract

**Introduction:**

Despite healthcare workers (HCWs) facing mental health challenges during COVID-19, their use of psychological support is limited. Understanding these dynamics is crucial for identifying mental health needs and vulnerable groups within Spanish healthcare services.

**Objectives:**

This study analyzes psychological support use among Spanish HCWs post-pandemic onset over 2 years, and its link to workplace and COVID-19 factors from 2020 data.

**Methods:**

Longitudinal research involved Spanish HCWs. Data from online surveys covered demographics (age and gender), depressive symptoms (PHQ-9), workplace-and COVID-19-related factors (type of job, direct exposure to COVID-19 patients, adequate access to personal protective equipment, social stigma for working with COVID-19 patients, decision making on patient prioritization, and perceived social network support at work), and psychological support use across 2020, 2021, and 2022. We received responses from 296, 294, and 251 participants at time points 1, 2, and 3, respectively.

**Results:**

Predominantly female participants (n=242, 82%) and with a median age of 43 years. Psychological support seeking increased from 15% in 2020 to 23% in 2022. Notably, one in four HCWs not seeking help showed major depressive disorder symptoms. Predictors for seeking support included patient prioritization decision-making (OR 5.59, 95% CI 2.47-12.63) and probable depression (wave 2: OR 1.12, 95% CI 1.06-1.19; wave 3: OR 1.10, 95% CI 1.04-1.16). Table 1 shows the association between workplace- and COVID-19-related variables at baseline and use of psychological support at follow-up.

**Table:** 

Table 1.				
	Wave 2		Wave 3	
	Odds ratio	95% CI	Odds ratio	95% CI
Direct exposure to COVID-19 patients	2.03	(0.93, 4.41)	1.03	(0.53, 2.03)
Access to protective equipment	0.81	(0.55, 1.19)	1.10	(0.75, 1.60)
Social stigma for working with COVID-19 patients	1.20	(0.84, 1.71)	1.13	(0.80, 1.60)
Patient prioritization	5.59	(2.47, 12.63)	1.45	(0.64, 3.26)
Social support from colleagues	1.45	(0.88, 2.41)	1.17	(0.75, 1.85)

**Conclusions:**

A significant portion of HCWs may seek psychological support post-pandemic, regardless of prior workplace or COVID-19 stressors. Targeted mental health initiatives are crucial, emphasizing health promotion, primary prevention, and support for individuals, including those with depressive symptoms.

**Disclosure of Interest:**

None Declared

